# Utilization and associated determinants of multi-sectoral approach in zoonotic disease surveillance among animal and human healthcare workers in Nakuru County, Kenya

**DOI:** 10.12688/openreseurope.17583.2

**Published:** 2024-09-02

**Authors:** Levi Cheptoyek, Gideon Kikuvi, John Gachohi

**Affiliations:** 1 P.O. Box 62000-00200, Jomo Kenyatta University of Agriculture and Technology, Nairobi, Kenya

**Keywords:** Multisectoral collaboration in zoonotic disease surveillance

## Abstract

**Background:**

Zoonoses are naturally transmissible between humans and animals. Globally, they account for more than 60% of human infections, 75% of emerging infections, 2.7 million human deaths, and 10% of the total DALYs lost yearly in Africa. In the last three decades, Kenya has had sporadic outbreaks of zoonoses. To increase the speed of reporting and efficiencies in detection and control, a multi-sectoral collaboration in zoonotic disease surveillance (MZDS) between human and animal health workers is essential. In an effort, Zoonotic disease unit (ZDU) in Kenya has been established at national and county levels.

**Methods:**

A cross sectional study was carried out to determine the level of utilization of multisectoral collaboration and its associated determinants in zoonotic disease surveillance among animal and human healthcare workers in Nakuru County. Quantitative data was gathered from 102 participants and qualitative data from 5 key informants. To test for significant differences, Chi-square and independent t-test were used.

**Results:**

MZDS utilization level was 16% and the factors associated with MZDS utilization include; knowing what MZDS entails, education level, sector affiliation, trainings, supportive infrastructure, budget allocation and data storage. Lack of financing and poor coordination are hindrances to MZDS.

**Conclusion:**

There is need to finance MZDS activities, strengthen coordination mechanisms, carry out more sensitization and trainings among animal and human healthcare workers.

## Introduction

Zoonoses are diseases that are naturally transmissible between humans and animals ^1^. Globally, they account for; more than 60% of human infections, about 75% of emerging infections, 2.7 million human deaths yearly, 10% of the total DALYs lost and wildlife is at the spotlight for outbreaks ^2– 6^. Human health and animal health are inextricably linked and thus multi-sectoral efforts in disease surveillance is required to detect and respond timely to zoonoses so as to reduce their burden ^7^.

In 2014, WHO and the United States jointly launched the Global Health Security Agenda (GHSA) which aims at building capacity for early detection and effective response to zoonoses through multisectoral collaboration ^8^. Some collaborative efforts have been established between universities and research institutions for example Afrique One Consortium in Chad, Ivory Coast, Tanzania, Uganda, and Senegal that fosters OH ^9^.

In 2012, Kenya established a Zoonotic Disease Unit (ZDU) to provide a multi-sectoral collaborative platform for better coordination of efforts geared towards bridging the gap between human and animal health in priority zoonoses prevention and control ^10^. The ZDU unit comprises of a medical epidemiologist deployed from MOH, a veterinary epidemiologist deployed from the Ministry of Agriculture, Livestock and Fisheries (MALF), data analyst and an administrative assistant. In 2013, following devolution, ZDU established county and sub county platforms and surveillance officers in both animal and human health trained ^4^ and in 2022, OH Strategic Plan for Prevention and Control of Zoonotic diseases in Kenya was launched ^11, 12^.

Multi-sectoral zoonotic disease prioritization with equal engagement from all sectors active in zoonoses surveillance is one of the most cost-effective ways to prevent, detect, and respond to public health threats ^13^. In Sub-Saharan Africa, only 14% of the countries have multi-sectoral action plan for zoonoses and 32% have established inter-sectoral zoonotic surveillance systems ^14^. The barriers towards effective MZDS are; lack of supportive policies, insufficient funding, conflicting departmental priorities and limited institutional capacities ^3, 15, 16^. Therefore, early disease detection and control at the sources is delayed and risk widespread outbreaks and mortalities in both animals and humans resulting in disruptions in trade, economic losses and strain on public health resources ^17^.

In the last three decades, Kenya has had sporadic outbreaks of priority zoonoses ^4^. In 2007, over 300 people died of RVF with livestock loses of over US$9.3 million ^18^. Nakuru county is a hotspot for Anthrax, Rabies and Brucellosis because of its proximity to wild life. Between 2014 and 2017, Anthrax infected 71 people with 2 deaths and over 700 animals died ^19^. To detect and control zoonoses at the source, a MZDS is essential ^13^. There is however very scanty information on utilization of MSDS in Nakuru County. The aim of this study was to determine the level of utilization of MZDS and its associated factors among animal and human healthcare workers in Nakuru county, Kenya.

## Materials and methods

### Type and period of study

Analytical cross-sectional study design was used and the study covered the period between August 20, 2023 and October 15, 2023.

### Setting of the study

The study was conducted in Nakuru County, a third most popular county and located in Rift valley region of Kenya. It is bordered by; Baringo, Laikipia, Nyandarua, Kajiado, Narok, Bomet and Kericho counties. It has an area of 7,496.5km², a population of 1,603,325 ^20^ and with physical features like; L. Naivasha a home of millions of flamingos, sanctuary to protect Rothschild giraffe and black rhinos, and Nakuru national park. The site is a hotspot for Anthrax, Brucellosis and Rabies ^21^ and this formed the basis for purposive selection of the study area.

### Study population

a. The human healthcare workers who included; county director of public health, county director of medical services, county data analyst, Emergency Operations Center coordinator and public health officers.
b. Animal health workers who included county director of veterinary services and animal health workers at subcounty level.

The public health officers and animal health workers (excluding the county director of veterinary services) served as frontline surveillance officers at subcounty levels. Public health officers were attached to sentinel sites for example public and health facilities including subcounty hospitals, abattoirs and butchers where they captured surveillance data. They also monitored air and water quality as well as the health of the physical environment like managing waste disposal. The animal health workers on the other hand were focal persons for disease surveillance from the animal side. They visited farms and offered extension and treatment services, inspect animals before and after slaughter, as well as receiving any animal related issues from the community. 

At county levels were the county directors who included; county director for veterinary services, county director for public health, county director of medical services, county Data analyst and county emergency and operations center officer. This team provided strategic direction for zoonoses preparedness, detection and response at the county government.

***Inclusion Criteria.*** Human and Animal Healthcare workers who consented.

***Exclusion Criteria.*** Animal and human healthcare workers who were not on duty over the period of the study and those who did not consent.

### Sampling

A census was conducted.

### Data collection and tools

A semi-structured pretested interviewer-administered questionnaire was used to collect quantitative data (demographic, awareness, attitudes and practices) from 102 frontline animal health workers and public health workers who served at sub-county levels. A key informant interview guide was used to collect data on institutional factors (funding, space, priorities, staffing, MZDS plans, political will) from county director of veterinary services, county director of public health, county director of medical services, county data analyst and county emergency and operations center coordinator. Audio tape recording was used to maintain and capture their exact words. The sessions lasted 45–60 minutes. Saturation marked the end of interview sessions. Three research assistants were recruited to administer the questionnaires and conduct the interviews with the participants face to face.

### Data processing and analysis

Quantitative data was entered to an excel file, cleaned and exported to R 4.3.1 Software for descriptive and inferential statistical analysis. The descriptive statistics included frequencies and percentages and were presented on tables. Hearing about MZDS and the regularity of carrying out joint data collection, analysis, interpretation and sharing with other sectors formed the basis for inferential statistics. Those who regularly (weekly, monthly or quarterly) carried out joint data collection, analysis, interpretation and sharing with other sectors were termed as, Utilizing MZDS. Utilization level was assessed through the other variables (demographic, awareness, attitudes and practices). Chi-square test was used to determine the association between demographic (age, gender, education, duration in service, sector) variables and utilization of MZDS. Independent t-test was used to measure the association between variables of awareness, attitudes and practices towards MZDS and MZDS utilization. Statistical significance was set at a p-value < 0.05 and 95% confidence interval. Qualitative data was analyzed manually using MS Excel spreadsheets. The transcripts were read again and again and listening to voice recordings repeatedly ^22^. This helped to understand the concept and the words, phrases, sentences even sections which labeled and coded ^23^. The concept to be coded depended on whether it was repeated several times, as the code would be so many, similar codes were grouped into categories which were used to generate themes ^24^. The themes and categories were the main results of the study.

### Ethical considerations

Ethical approval and a research permit were attained from: the ethics review board for Jomo Kenyatta University of Agriculture and Technology (JKUAT), Ref: JKU/ISERC/023/0842, JKUAT Board of postgraduate studies, Ref: JKU/2/11/HSH315R-0088/2022, National Council for Science, Technology and Innovation, Ref: NACOSTI/P/23/25534, and Nakuru county. Written informed consent was acquired from the participants. Completed questionnaires were kept under lock and key and accessed by only authorized people. Soft copies and all analyzed data were passworded.

## Results

A total of 102 animal and public health surveillance officers who served as frontline surveillance persons, and five (05) key informants were consented and enrolled to the study. The respondents interviewed were 100% of the targeted population.

### Demographic characteristics of respondents

From the 102 frontline surveillance workers, majority (65%,N = 66) were males. Most were aged between 51–60 years (36.3%, N = 37). The mean duration in service was 17.41 ±9.86 years. More than forty percent had diploma as their highest education (46.1%, N = 47). And 78.4% (N = 80) were animal health workers while 21.6% (N =22) were public health officers. Other details are on Table 1 below.

**Table 1.** Demographic characteristics for the participants.

Variable	Frequency (N = 102)	Percent (%)
Gender		
Female	36	35.0
Male	66	65.0
Age		
20–30	18	17.6
31–40	29	28.4
41–50	18	17.6
51–60	37	36.3
Time in service		
Mean - 17.41, Minimum – 3, SD = 9.86, Maximum – 34		
Education level		
Certificate	30	29.4
Diploma	47	46.1
Undergraduate	21	20.6
Postgraduate	4	3.9
Sector		
Animal health	80	78.4
Human health	22	21.6

### Level of utilization of MZDS

Of the 102 participants, 73.5% (N=75) had heard about MZDS, among whom the level of utilization was 16% (N=12/75) and these were participants who frequently carried out joint data collection, analysis, interpretation and sharing with other sectors. The rest 84% (N=63/75) had either never utilized or only utilized it during outbreaks. A higher proportion of females (17.4%, 4/23) were involved in MZDS as compared to males (15.4%, 8/52). Knowing what MZDS entailed according to those who had heard about it had a higher utilization level (28.6%, 8/28) compared to those who did not know (8.5%, 4/47). Individuals with certificate level of education had no utilization (0%, 0/22) just like those with postgraduate (0%, 0/4), those with undergraduate had the highest utilization level of (29.4%, 5/17) followed by those with diploma (21.9%, 7/32). Participants who did not know of any organizational structure required for MZDS had no utilization (0%, 0/22), those who noted that a Subcounty one health committee, community health volunteers and the community had the highest utilization level (30%, 3/10) followed by those who noted that no particular structure is required had utilization level of (20.9, 9/43). Participants who noted that requiring a zoonotic disease outbreak for having a functional MZDS had the highest utilization level (66.7%, 2/3) followed by those who noted that requiring Commitment and participation by all sectors (37.5%, 6/16), then those who noted trained staff (20%, 1/5) and lastly those who noted budget, trained staff and office space (11.5%, 3/26). Respondents with frequent budgetary allocation had the highest utilization level (66.7%, 2/3) followed by those who have never had any budgetary allocation (44.4%, 4/9) and those who have never had any budgetary allocation with the lowest utilization level (9.5%, 6/57). Other utilization level for other variables is shown on the Table 2, below.

**Table 2.** Level of utilization of MZDS.

Variable		Level of Utilization of MZDS	
		Utilized (16%, N = 12)	Didn’t utilize (84%, N = 63)
Demographic Characteristics			
Age	20–30	1 (12.5%)	7 (87.5%)
	31–40	4 (18.2%)	18 (81.8%)
	41–50	4 (26.7%)	11 (73.3%)
	51–60	3 (10.0%)	27 (90.0%)
Sector	Animal health	5 (9.4%)	48 (90.6%)
	Public health	7 (31.8%)	15 (68.2%)
Awareness on MZDS			
Respondents who had heard about of (MZDS)		12 (16%)	63 (84%)
Respondents who listed the five priority- zoonotic diseases	Correct	2 (25%)	6 (75%)
	Incorrect	10 (14.9%)	57 (85.1%)
Ranking priority zoonoses in-terms of severity, epidemic & socio-economic burden	Correct	1 (33.3%)	2 (66.7%)
	Incorrect	1 (15.3%)	61 (84.7%
Attitudes			
MZDS is important for early disease detection & response	Agree	12 (16%)	63 (84%)
I am an important actor for MZDS	Agree	12 (16.2%)	62 (83.8%)
	Neutral	0 (0)	1 (100%)
Sensitization on MZDS by devolved	Agree	6 (50%)	6 (50%)
County ZDU is adequate	Neutral	4 (8.5%)	43 (91.5%)
	Don’t Agree	2 (12.5%)	14 (87.5%)
With initiation of MZDS, infrastructures for zoonoses surveillance has improved	Agree	10 (41.7%)	14 (58.3%)
	Neutral	2 (6.1%)	31 (93.9%)
	Don’t Agree	0 (0)	18 (100%)
Results and information on MZDS is adequately shared to all sectors	Agree	6 (37.5)	10(62.5%)
	Neutral	3 (7%)	40 (93%)
	Don’t Agree	3 (18.8%)	13 (81.3%)
Practice			
Proportion of Respondents who received information on MZDS	Never	1 (12.5%)	7 (87.5%)
	Rarely	5 (11.4%)	39 (88.6%)
	Frequently	6 (26.1%)	17 (73.9%)
Participation in any training on MZDS	Never	3 (15.8%)	16 (84.2%)
	Rarely	4 (8.2%)	45 (91.8%)
	Frequently	5 (71.4%)	2 (28.6%)
Regularity of storing data and information on MZDS in your sub-county (N = 51)	Never	0 (0)	14 (100%)
	Rarely	4 (8.9%)	41 (91.1%)
	Frequently	8 (50%)	8 (50%)
Proportion of respondents who notify a health counterpart in the sub-county	Frequently	12 (17.1%)	58 (82.9%)
	Rarely	0 (0)	5 (100%)
Community involvement in zoonoses surveillance activities.	Never	0 (0)	4 (100%)
	Rarely	1 (11.1%)	8 (88.9%)
	Frequently	11 (17.7%)	51 (82.3%)

### Demographic factors associated with utilization of MZDS

Education level (χ =3.889, df = 3, p = 0.026) (χ =3.889, df = 3, p = 0.026) and sector (χ =1.657, df = 3, p = 0.023) were significantly associated with utilization of MZDS. Individuals with diploma and undergraduate education were more likely involved in MZDS as opposed to those with certificate and postgraduate education. Individuals from public health were also more likely involved as opposed to individuals from animal health. All other variables considered in this study are shown on Table 3, below.

**Table 3.** Association between demographic variables and utilization of MZDS.

Variable	Utilized MZDS (16%)	Didn’t utilize MZDS (84%)	Chi-square value	Df	p-value
Gender					
Female (n=23)	4(17.4%)	19 (82.6%)			
Male (n=52)	8 (15.4%)	44 (84.6%)	0.997	1	0.318
Age					
20-30	1 (12.5%)	7 (87.5%)			
31-40	4 (18.2%)	18 (81.8%)			
41-50	4 (26.7%)	11 (73.3%)			
51-60	3 (10.0%)	27 (90.0%)	2.200	3	0.520
Education level					
Certificate	0 (0)	22 (100%)			
Diploma	7 (21.9%)	25 (78.1%)			
Undergraduate	5 (29.4%)	12 (70.6%)			
Postgraduate	0 (0)	4 (100%)	3.986	3	0.026
Sector					
Animal health	5 (9.4%)	48 (90.6%)			
Public health	7 (31.8%)	15 (68.2%)	1.657	1	0.026
Time spent in service	16.08	19.81	1.206	73	0.232

### Association between awareness, attitude and practices towards MZDS and utilization of MZDS

Utilization of MZDS was associated with being awareness of what it entails (t= 2.269, df = 15.05, p = 0.038). Participants who utilized MZDS had a higher mean score (mean= 0.6825) than those who did not utilize (mean= 0.3333). Agreeing that that sensitization on MZDS by devolved County Zoonotic Disease Unit (ZDU) was adequate (t = 2.466, df = 73, p = 0.016) was significantly associated with utilization of MZDS. Those who had never engaged in joint work had a higher mean score (2.333), which leans towards disagreeing than those who engaged on joint work (1.923). Individuals who had been trained on MZDS had a higher utilization level (t=2.227, df = 3, p = 0.029). Those who believed that infrastructure for MZDS has improved following devolution (t= 4.209, df= 73, p = 0.001) also had a higher utilization level. Having a budget and storing data on MZDS were associated with higher utilization levels. Other factors were not significant as shown on Table 4, below.

**Table 4.** Association between awareness, attitudes and practices towards MZDS and utilization of MZDS.

Variable	Utilized MZDS (16%)	Didn’t utilize MZDS (84%)	t-test	Df	p-value
**Awareness**					
Organizational structure of MSDS at subcounty?					
Correct	3 (4.0%)	7 (9.3%)			
Incorrect	9 (12.0%)	56 (74.7%)	-1.017	3.132	0.327
Respondents who correctly listed the five priority zoonotic diseases in Kenya					
Correct	2 (25%)	6 (75%)			
Incorrect	10 (14.9%)	57 (85.1%)	-0.728	3	0.469
Ranking priority zoonoses in-terms of severity, epidemic potential & socio-economic burden					
Correct	1 (33.3%)	2 (66.7%)			
Incorrect	11 (15.3%)	61 (84.7%)	-0.829	3	0.410
Requirements for MZDS to be functional					
Correct	12 (16%)	60 (80%)			
Incorrect	0 (0%)	3 (4.0%)	-1.762	2	0.083
**Attitude**					
I am an important actor in Multisectoral approach					
Agree	12 (16.2%)	62 (83.8%)			
Neutral	0 (0)	1 (100%)	0.434	73	0.666
Multisectoral approach has easily been implemented in my area of work.					
Agree	7 (29.2%)	17 (70.8%)			
Neutral	3 (7.3%)	38 (92.7%)			
Don’t Agree	2 (20%)	8 (80%)	1.342	73	0.184
Information on MZDS is adequately shared to all sectors					
Agree	6 (37.5%)	10 (62.5%)			
Neutral	3 (7%)	40 (93%)			
Don’t Agree	3 (18.8%)	13 (81.3%)	1.448	73	0.152
**Practices**					
Proportion of Respondents who received information on MZDS					
Never	1 (12.5%)	7 (87.5%)			
Rarely	5 (11.4%)	39 (88.6%)			
Frequently	6 (26.1%)	17 (73.9%)	-1.339	3	0.185
Regularity of storing data and information on MZDS					
Never	0 (0)	14 (100%)			
Rarely	4 (8.9%)	41 (91.1%)			
Frequently	8 (50%)	8 (50%)	-4.211	3	0.001
Budget for MZDS in the subcounty					
Never	6 (8.0%)	57 (76.0%)			
Rarely	4 (5.3%)	5 (6.7%)			
Frequently	2 (2.7%)	1 (1.3%)	-2.422	11.9	0.032

### Institutional factors associated with utilization of MZDS

Results from the qualitative data indicated that there was no; proper organizational structure, budgetary allocation and infrastructure for MZDS. One key informant argued that “ *… multi-sectoral collaboration has no separate resources, departments use their own routine resources e.g., staff, vehicles, computers, emails and budget”.* On organizational structure, there were no goals, workplans or policy frameworks on MZDS. one interviewee reported that *“… there is no well-defined organization structure, however, the directors of the two departments take the lead”.* On political support, MZDS was still more technical and political support was very low. On suggestions on how to improve MZDS, there is need to provide a budgetary allocation and capacity building. *One argued that “… there is need for proper budgetary allocation and support for technical group”.*

## Discussion 

### The Level of MZDS among animal and human health care workers in Nakuru County

The level of MZDS was 16%, which is low in a zoonotic prone area. The rate reported in other sub-Saharan countries with multisectoral collaborations is 32% ^14^ which is higher compared to findings of our study. However, Munyua *et al.*, 2019, noted that MZDS in Kenya are generally low due to limited funding and competing priorities. This study further identified other factors like awareness, education level, sensitization and trainings, sector where participants belong and data storage as other factors were associated with the low utilization. Given that MZDS is key for early zoonotic disease detection and response and that the overall utilization level is less than twenty percent, the initiative should be addressed much more seriously by all sectors to enhance timely sharing of zoonotic information ^25^.

### Demographic characteristics associated with MZDS

Nakuru county animal and health staff benefit from the Field Epidemiology Training Program (FETP) which is a postgraduate training and it’s likely that it was a motivation for higher utilization amongst those with undergrade training. Human health workers were more likely to involved in collaboration which deviates from the previously reported evidence by 26 who noted that animal health workers were twice more likely to collaborate compared to human health workers. The deviation may be because trainings on collaboration were born from COVID-19 outbreak and the lead team was human health. Human health has partners like Africa Medical Research Foundation (AMREF), CDC who fund their workplans. Since animal health workers are directly in contact with animals, there is great need to bridge the gaps of their MZDS under-utilization.

### Awareness, attitude and practice factors associated with MZDS

Knowing what MZDS entails was associated with higher utilization however, less than a half of those who knew what MZDS entails utilized it. This might be explained by the fact that was no budgetary allocation for collaboration activities at the county and so no accountability. Sensitization on MZDS by devolved County ZDU was found to be adequate and associated with higher utilization of MZDS. Those who had ever trained in MZDS were more likely to utilize MZDS and Infrastructure for MZDS had improved following devolution. 27 noted that awareness of health and veterinary departments was very key for MZDS. 15 also reported that knowledge attained from trainings had significant effect on utilization of MZDS. Nakuru county has partners like AMREF and ZDU which occasionally offer trainings and sensitization to the animal and healthcare workers in Nakuru county with focus on bi-sectoral collaboration at sub-county level using Community Event-Based Surveillance System (CEBs) ^28^. With only 10% frequently receiving trainings and the rest either never or only been trained once in the last three years, it is possible to increase individual participations in MZDS by stepping up trainings. Data storage by participants was associated with MZDS as well as budget allocation. This could be explained by the fact that trainings and sensitizations provide an avenue for participants to store data and having a budget facilitates accomplishing routine MZDS activities.

### Institutional factors for MZDS

There is no financing for MZDS and as reported by 16, Kenya’s challenges for MZDS are insufficient funding and competing priorities. Sectors in cooperate MZDS activities along their sector workplans and since there is no particular budget and accountability, less can be devoted to MZDS. There were no MZDS goals, workplans and organizational structure and this is in line with the findings of 26 who reported lack of plans and networks by institutions are limiting inter-sectoral collaborations. There also exists more of informal communications between sectors for example, visits, phone calls are predominantly used for information sharing. These findings agree with 25 who noted that lack of effective coordination and commitment to OH preparedness and response efforts, lack of an agreed mechanism for sharing information across sectors and partners were key hindrances to MZDS.

## Limitations

Being a cross-sectional study, the association from the subset analysis cannot be assumed to mean cause-effect relationship but rather an indicator of factors that are commonly identified with the comparative sub-groups. Another limitation, no confirmation was done whether indeed the respondents utilized the MZDZ, thus totally relying respondents’ sincerity.

## Conclusion

MZDS should be a major issue of concern in sub-Saharan Africa for early disease detection. Zoonoses can aggravate fast causing a myriad of sequels, and hence the need for early detection, timely response and control. From this study, there is a low level of MZDS in Nakuru County. The factors associated with utilization of MZDS included; sector, education level, data storage, sensitization and trainings as well as lack of proper coordination, organizational and financing mechanisms.

## Recommendations

The National government and the county government of Nakuru need to pay more attention towards MZDS activities through financing MZDS activities, sensitization and training staff. There is also need to provide physical infrastructure like mobility means, office space and its infrastructure as well as provide information and communication technology for MZDS.

## Data Availability

Dryad: Data from: Multisectoral approach in zoonotic disease surveillance, https://doi.org/10.5061/dryad.g1jwstqzm ^29^. This project contains the following underlying data: MZDSCodes.csv MZDZ2.csv Data are available under the terms of the Creative Commons Zero "No rights reserved" data waiver (CC0 1.0 Public domain dedication).
