# Supplementary material for: Utilization and associated determinants of multi-sectoral approach in zoonotic disease surveillance among animal and human healthcare workers in Nakuru County, Kenya
Source: Open Res Eur. 2024 Sep 2;4:103. Originally published 2024 May 28. [Version 2] doi: 10.12688/openreseurope.17583.2 (PMC11607475; doi:10.12688/openreseurope.17583.2)
Supplement: Peer review history

# Reviewer report: Reviewer response for version 2

**DOI:** 10.21956/openreseurope.19948.r47268

**Published:** 2024-11-29

**Authors:** Nick Wheelhouse

**Affiliations:** 1 Edinburgh Napier University, Edinburgh, Scotland, UK

The article reports on the utilization of multi-sectoral collaboration in zoonotic disease surveillance among animal and human healthcare workers in Nakuru County, The paper does provide some key insight into potential issues around training and MZDZ awareness within the participants. This is a a limited study focusing on key frontline workers and the population is relatively small but the work has been carried out and presented in a solid fashion and there are important take home messages. Previous reviewers highlighted a number of issues in previous drafts but these appear to have been adequately addressed.

Is the study design appropriate and does the work have academic merit?

Yes

Is the work clearly and accurately presented and does it cite the current literature?

Yes

If applicable, is the statistical analysis and its interpretation appropriate?

Yes

Are all the source data underlying the results available to ensure full reproducibility?

Yes

Are the conclusions drawn adequately supported by the results?

Yes

Are sufficient details of methods and analysis provided to allow replication by others?

Yes

Reviewer Expertise:

Zoonoses, Veterinary microbiology

I confirm that I have read this submission and believe that I have an appropriate level of expertise to confirm that it is of an acceptable scientific standard.

# Reviewer report: Reviewer response for version 2

**DOI:** 10.21956/openreseurope.19948.r43893

**Published:** 2024-09-23

**Authors:** Jolly Musoke

**Affiliations:** 1 University of the Free State, Bloemfontein, Free State, South Africa

I have reviewed the revision. 

 I am happy with the amendments. 

 Please proceed and accept.

Is the study design appropriate and does the work have academic merit?

Partly

Is the work clearly and accurately presented and does it cite the current literature?

Partly

If applicable, is the statistical analysis and its interpretation appropriate?

I cannot comment. A qualified statistician is required.

Are all the source data underlying the results available to ensure full reproducibility?

Yes

Are the conclusions drawn adequately supported by the results?

Yes

Are sufficient details of methods and analysis provided to allow replication by others?

Partly

Reviewer Expertise:

Zoonoses, Medical Microbiology

I confirm that I have read this submission and believe that I have an appropriate level of expertise to confirm that it is of an acceptable scientific standard.

# Reviewer report: Reviewer response for version 1

**DOI:** 10.21956/openreseurope.18999.r41564

**Published:** 2024-08-08

**Authors:** Budi Utomo

**Affiliations:** 1 Universitas Airlangga, Surabaya, East Java, Indonesia

The writing is clear and incorporates recent literature, though some references are over ten years old. The study design is appropriate, and the work has practical merit. A mixed-methods approach is recommended: quantitative for general respondents and qualitative for key informants, such as community leaders and district health officials.

 However, the details of the method and data analysis are unclear, particularly regarding which variables use the 2-sample t-test and which use the chi-square test, making it difficult to replicate this research; especially in the aspect of information retrieval in key persons, there is no specific characteristic information.

 Your sentence is mostly clear, but here’s a slightly refined version for clarity:

 "Corrections are needed both methodologically and in statistical studies to ensure that the findings can be easily understood, especially by those who are experts in statistics and research methodology."

 A conclusion can be considered valid if it meets the methodological requirements and adheres to statistical standards.

Is the study design appropriate and does the work have academic merit?

Partly

Is the work clearly and accurately presented and does it cite the current literature?

Yes

If applicable, is the statistical analysis and its interpretation appropriate?

Partly

Are all the source data underlying the results available to ensure full reproducibility?

Partly

Are the conclusions drawn adequately supported by the results?

Partly

Are sufficient details of methods and analysis provided to allow replication by others?

Yes

Reviewer Expertise:

My area of expertise encompasses methodological, statistical, and medical epidemiology

I confirm that I have read this submission and believe that I have an appropriate level of expertise to confirm that it is of an acceptable scientific standard, however I have significant reservations, as outlined above.

# Reviewer report: Reviewer response for version 1

**DOI:** 10.21956/openreseurope.18999.r41561

**Published:** 2024-07-23

**Authors:** Jolly Musoke

**Affiliations:** 1 University of the Free State, Bloemfontein, Free State, South Africa

The article aims to report on the utilization of MZDS among animal and human healthcare workers in Nakuru County, Kenya. My main concern with the article is that the interviewed participants do not represent all key stakeholders of the MZDS. In my opinion, including medical officers, veterinary officers, and policymakers would have provided a more comprehensive representation rather than focusing solely on the “frontline workers”. It was unclear to me if the heads of veterinary services, public health, and medical services were included, as participants as stated in the data collection and tools section. The terms "public health" and "human healthcare workers" were used interchangeably, though they have different meanings. Please clarify which officers were included.

 Language editing is required, particularly under the results subsection "level of utilization of MZDS," as it was confusing to understand. The structure and flow of information need to be revisited. The results presented in tables are duplicated, with and without statistical analysis. I recommend presenting the results only once, with statistical analysis included, i.e., exclude Table 1 and Table 2.

 In text the authors reference using numbers may I suggest the use of the names of the referenced work rather than numbers.

 Unfortunately, the findings of this study add limited new knowledge to the literature, hence my opinion to not index the article.

Is the study design appropriate and does the work have academic merit?

Partly

Is the work clearly and accurately presented and does it cite the current literature?

Partly

If applicable, is the statistical analysis and its interpretation appropriate?

I cannot comment. A qualified statistician is required.

Are all the source data underlying the results available to ensure full reproducibility?

Yes

Are the conclusions drawn adequately supported by the results?

Yes

Are sufficient details of methods and analysis provided to allow replication by others?

Partly

Reviewer Expertise:

Zoonoses, Medical Microbiology

I confirm that I have read this submission and believe that I have an appropriate level of expertise to state that I do not consider it to be of an acceptable scientific standard, for reasons outlined above.

# Response

**Published:** 2024-08-23

**Authors:** Levi Cheptoyek

**Affiliations:** Environmental Health and Disease Control, Jomo Kenyatta University of Agriculture and Technology, Juja, Kiambu, Kenya

Thank you very much for taking time to review my work. It is a honor. I actually had to revist my methodology and together with my working team (supervisors) made it more clearer. May you kindly accept the responses to the comments. **Why not include medical officers and policy makers:**

1. Medical workers: Public health officers were attached to health facilities where clinicians like medical and clinical officers work. As the clinicians did clinical work, the surveillance focal persons looked through all the medical records and collected all the relevant information for reporting. For example, information on dog bites may not be brought to the attention of medical officers yet recorded by nurses in the outpatient department registers. So not much was lost by not including clinicians as surveillance focal persons provided all the information even up to the community levels.
2. Policy makers: On institutional factors, the main focus was on political will. This was captured from the key informants who included; county head of veterinary services, county head of public health, county director of medical services, county Data analyst and county emergency and operations center officer. For example, all the directors noted that there was low political will in allocating funds on multisectoral approach on zoonotic disease surveillance. Among the reasons for hesitance was competing priorities. Probably future research should unearth other factors for the hesitance.

 
**Clarification on the terms "public health" and "human healthcare workers" that were used interchangeably, though they have different meanings.** The human healthcare workers implied from the research topic referred to officers who were from the health sector. They included; county director of public health, county data analyst, county director of medical services, emergency operations center coordinator and public health officers. The study population has been made more clear. **Language editing is required, particularly under the results subsection "level of utilization of MZDS," as it was confusing to understand.** The language has been edited further. More detail on data collection, tools and how analysis was done has been clarified and there is now a flow with the reuslts presented. **The structure and flow of information need to be revisited. The results presented in tables are duplicated, with and without statistical analysis. ** The tables provide more detail not explained in the text. The team felt that the little text would help in interpreting what is contained in the tables. Statistical analysis was done for all the study variables. **In text the authors reference using numbers may I suggest the use of the names of the referenced work rather than numbers: ** Open Research Journal prefer use of hyper-linked numbers in referencing. **Unfortunately, the findings of this study add limited new knowledge to the literature, hence my opinion to not index the article: ** The government of Kenya established a zoonotic disease unit at the national level and cascaded the approach to county and subcounty levels. Studies available provide very limited information on the level of utilization of multisectoral approach by frontline staff whose duties has surveillance as one of their deliverables. There is very scanty information on individual level factors associated with utilization. The existing information focusses more on stating institutional factors hindering utilization. The team therefore believes that there is a thing to learn and build on from this study hence worthy indexing.
